# Secular changes in dementia risk indices among 70-year-olds: a comparison of two Finnish cohorts born 20 years apart

**DOI:** 10.1007/s40520-019-01204-8

**Published:** 2019-05-04

**Authors:** Jenni Vire, Marika Salminen, Paula Viikari, Tero Vahlberg, Seija Arve, Matti Viitanen, Laura Viikari

**Affiliations:** 1 Department of Geriatrics, Faculty of Medicine, Turku City Hospital, University of Turku, Kunnallissairaalantie 20, 20700 Turku, Finland; 2 City of Turku, Welfare Division, Yliopistonkatu 30, 20101 Turku, Finland; 3 Unit of Family Medicine, Faculty of Medicine, University of Turku, Lemminkäisenkatu, 20014 Turku, Finland; 4 Institute of Clinical Medicine, Biostatistics, University of Turku, Turku, Finland; 5 Division of Clinical Geriatrics Karolinska Institutet, Karolinska University Hospital, Huddinge, 14186 Stockholm, Sweden

**Keywords:** Dementia, Risk index, Older adults, Cohort comparison

## Abstract

**Aims:**

To compare dementia risk indices among two separate cohorts of 70-year-olds born 20 year apart.

**Methods:**

Community-dwelling 70-year-old Finns were examined with similar examinations in 1991 (*n* = 1032) and in 2011 (*n* = 960). Dementia risk was assessed with the CAIDE Dementia Risk Score (CAIDE) (*n* = 1516), the Brief Dementia Risk Index (BDRI) (*n* = 1598) and the Dementia Screening Indicator (DSI) (*n* = 1462).

**Results:**

The proportion of subjects with moderate or high risk for dementia was significantly higher in earlier than in later born cohort according to CAIDE (99% and 94%, respectively, *p* < 0.001) and BDRI (41% and 15%, *p* < 0.001), but not according to DSI (5% and 6%, *p *= 0.184). The total scores of the earlier born cohort were significantly higher than those of the later born cohort according to all three indices.

**Conclusions:**

According to dementia risk indices, it seems that dementia risk has decreased among community-dwelling 70-year-old subjects during the last decades in Finland.

## Introduction

Dementia is a major cause of institutionalization in older people, and therefore, it is a major global public health concern [1]. Although no curative treatment is available, epidemiological studies have provided evidence of modifiable risk and protective factors of dementia [2, 3]. Dementia risk indices are tools that quickly and efficiently combine information on known risk factors of dementia, and thus identify individuals with a risk for dementia who can be referred for more frequent monitoring and early interventions in order to prevent or delay onset of cognitive decline [4]. The aim of this cross-sectional study was to compare dementia risk indices in two birth cohorts of 70-year-old community-dwelling Finns born 20 years apart.

## Material and methods

S*tudy population*

The data of two 70-year-old cohorts of community-dwelling older people born in 1920 and 1940 and living in the city of Turku, in Southwest Finland, were collected by using similar postal questionnaires, interviews and clinical examinations in 1991 (The Turku Elderly Study) and 2011 (The New Turku Elderly Study). The data on the subjects included in both cohorts were obtained from the central population register. Altogether 1032 subjects from the earlier cohort (70% of those invited) and 960 subjects from the later cohort (73% of those invited) returned the postal questionnaires and were considered for inclusion in this study. The protocol of data collection and flow charts of the studies are described in detail previously [5]. Participants with missing data of dementia risk indices were excluded.

### Dementia Risk indices

The CAIDE Dementia Risk Score (CAIDE) [6], the Brief Dementia Risk Index (BDRI) [7] and the Dementia Screening Indicator (DSI) [8] were used to compare dementia risk between the cohorts. Slightly modified versions of both CAIDE and BDRI were used; DSI was used as an original.

The CAIDE, a seven-item risk index (range 0–15), includes age, education, gender, blood pressure, body mass index (BMI), total cholesterol, and physical activity [6]. In our study, physical inactivity was defined as not having daily outdoor activities. Those scoring ≥ 6 points have shown to have an elevated risk for developing dementia during the following 20 years among Finnish middle aged (39–64 years) population [6].

The BDRI [7] consists of 12 items (range 0–14): age, recall of three words presented after a brief delay, copying a figure of two pentagons that intersect to form a diamond, performing either of the first two steps of three-step request, naming at least ten four-legged animals in 30 s, self-reported “trouble keeping my mind on what I was doing” three or more days per week during the past month, medical history of stroke, peripheral artery disease or coronary artery bypass surgery, body mass index and alcohol consumption. We replaced the original item “naming ten four-legged animals in 30 s” with a mathematic exercise “serial sevens” included in Mini-Mental State Examination. We also included angioplasty in coronary artery bypass surgery. Older subjects (aged ≥ 65 years) with total scores of 0–2, 3–5 and ≥ 6 have previously been categorized as having a low, moderate, or high risk for developing dementia during a 6-year follow-up, respectively [7].

The DSI, designed specifically for usage in primary care settings in order to identifying older patients with an increased risk of dementia, includes the following seven items: age, educational attainment, body mass index, presence of diabetes mellitus, history of stroke, need for help in managing money or medications, and depressive symptoms (range 0–56). Subjects scoring ≥ 22 points have been classified as having a high risk for dementia in 65- to 79-year-olds during a 6-year follow-up [8].

### Ethics

The study protocol was approved by the City of Turku ethical committee on health care and the ethical committee of the Hospital district of Southwest Finland. Informed consent was obtained from all participants.

### Statistical analyses

Differences in dementia risk items and categorized dementia risk level between two cohorts were analyzed by using the Chi-squared test and Fisher’s exact test. Differences in mean scores of indices were tested by using two-sample *t* test. For BDRI and DSI, analyses were also conducted separately for men and women because gender was not included in those indices. *p *values < 0.05 were considered statistically significant. All statistical analyses were performed using SAS System for Windows, version 9.4 (SAS Institute Inc., Cary, NC, USA).

## Results

Altogether, 1516, 1598 and 1462 participants (63% of women) were included in the comparison of dementia risk with CAIDE, BDRI, and DSI between the cohorts, respectively (Table 1). The proportion of subjects with an increased risk for developing dementia was significantly higher in 1920 cohort compared to 1940 cohort according to categorized CAIDE and BDRI but not according to that of DSI. There were distinct differences in proportions of subjects (cohorts combined) categorized as having an increased (moderate or high) risk for developing dementia according to three indices being 96%, 27% and 6% according to CAIDE, BDRI, and DSI, respectively. The total risk scores of the earlier born cohort were significantly higher than those of the later born cohort according to all three indices.

**Table 1:** Characteristics of CAIDE Dementia Risk Score, the Brief Dementia Risk Index and the Dementia Screening Indicator in 1920 cohort (*n* = 1032) and 1940 cohort (*n* = 956)

	Points	1920 cohort *n* = 719 *n* (%)	1940 cohort *n* = 797 *n* (%)	*p* value
CAIDE Dementia Risk Score (CAIDE)				
Age > 53 years	4	719 (100)	797 (100)	1.000
Education (years)				< 0.001
≥ 10	0	50 (7)	169 (21)	
7–9	2	88 (14)	183 (23)	
< 7	3	570 (79)	445 (56)	
Male	1	230 (32)	328 (41)	< 0.001
Systolic blood pressure > 140 mm Hg	2	513 (71)	516 (65)	0.006
Body mass index > 30 kg/m^2^	2	115 (16)	194 (24)	< 0.001
Total cholesterol > 6.5 mmol/L	2	241 (34)	80 (10)	< 0.001
Physical inactivity	1	35 (5)	27 (3)	0.143
Increased risk for dementia	≥ 6	711 (99)	746 (94)	< 0.001
Total score, mean (SD)		9.39 (1.75)	8.53 (1.99)	< 0.001
The Brief Dementia Risk Index (BDRI)		*n* = 704	*n* = 894	
Age < 75 years	0	704 (100)	894 (100)	1.000
Delayed recall, < 2 of 3 words	2	323 (46)	96 (11)	< 0.001
Incorrectly copying intersecting pentagons	1	100 (14)	56 (6)	< 0.001
Incorrectly taking or folding a paper	1	9 (1)	5 (1)	0.176
Serial seven^a^, < 3 of 5 correct	1	73 (10)	82 (9)	0.422
Self-reported ‘trouble keeping mind on things’ often or almost always	1	131 (19)	154 (17)	0.474
Stroke	1	71 (10)	80 (9)	0.441
Peripheral artery disease	1	55 (8)	16 (2)	< 0.001
Coronary artery bypass surgery^b^	1	9 (1)	49 (5)	< 0.001
Body mass index < 18.5 kg/m^2^	1	7 (1)	6 (1)	0.578
Lack of current alcohol consumption	1	447 (63)	743 (83)	< 0.001
Risk level according to BDRI				< 0.001
Low	0–2	415 (59)	759 (85)	
Moderate	3–5	282 (40)	128 (14)	
High	≥ 6	7 (1)	7 (1)	
Total score, mean (SD)		2.20 (1.35)	1.55 (1.07)	< 0.001
The Dementia Screening Indicator (DSI)		*n* = 631	*n* = 831	
Age of 70 years	5	631 (100)	831 (100)	1.000
Less than 12 years of education	9	590 (94)	644 (78)	< 0.001
Body mass index < 18.5 kg/m^2^	8	5 (1)	6 (1)	1.000
Type 2 diabetes	3	76 (12)	138 (17)	0.015
Stroke	6	67 (11)	77 (9)	0.390
Need for help in managing money or medications	10	16 (3)	25 (3)	0.588
Depressive symptoms^c^	6	30 (5)	76 (9)	0.001
Increased risk for dementia	≥ 22	30 (5)	53 (6)	0.184
Total score, mean (SD)		15.02 (3.94)	13.94 (5.23)	< 0.001

^a^A mathematic exercise to replace the original characteristic (“Inability to name 10 four-legged animals in 30 s”) of the index

^b^Includes also angioplasty

^c^Use of anti-depressant medications or self-rated feelings of depression

BDRI and DSI was also analyzed separately in women and men, because gender was not included in either indices. According to BDRI, 38% and 9% (*p* < 0.001) of women in 1920 and 1940 cohort, respectively, had moderate or high risk for dementia; corresponding proportions of men were 47% and 23% (*p* < 0.001). Also the total scores of BDRI were significantly higher in 1920 cohort compared to 1940 cohort both in women (Mean ± standard deviation 2.10 ± 1.36 and 1.35 ± 0.92, respectively) (*p* < 0.001) and in men (2.41 ± 1.30 and 1.84 ± 1.20) (*p* < 0.001). According to DSI, significant difference was found only in total scores among women (15.06 ± 3.56 and 13.68 ± 5.16) (*p *≤ 0.001), and the difference was in favor of the later cohort.

## Discussion

The results of our study showed that dementia risk, assessed by using dementia risk indices, has decreased among Finnish community-dwelling older adults during the last decades. This is consistent with the evidence of decreasing age-specific incidence and stable or decreasing age-specific prevalence of dementia in Europe [9-11]. Explanations for the decreasing incidence of dementia are suggested to be higher education [12, 13], brain-healthy lifestyle, better treatment of major vascular risk factors [12, 14], better access to health care interventions as well as improvements in living conditions and social welfare in successive cohorts [15]. Despite time trends in occurrence of dementia, the number of people with dementia is projected to increase mainly because of increased life expectancy and declining rates of mortality [10].

In our study, it was notable that proportions of subjects with an elevated risk for dementia varied a lot based on the index used being distinctly highest according to CAIDE. CAIDE was originally developed for a middle aged population [6], and it showed poor performance in our study which is in line with previous studies also conducted among elderly populations [16-20]. In our study population, all participants received the highest score for age. In addition, CAIDE highlights the role of vascular factors which have found to have inverse associations with dementia among older age groups [20-22]. Therefore, CAIDE is a good index for mid-life dementia risk prediction [23] but application of it among older adults is limited. However, in the Finnish population-based CAIDE study, a late-life dementia index has recently been developed by using a supervised machine learning method which is able to handle large amounts of data, structure risk factors into groups and give a comprehensive overview of an individual’s predictive profile pointing the most relevant risk factors. This late-life dementia index could be useful for dementia prediction of older adults in research settings [10].

BDRI [7] and DSI [8], used in our study, are validated among older ( ≥ 65 years of age) population, and could, therefore, be appropriate instruments to identify older subjects with an elevated risk for developing dementia later in life. Both indices are brief and easy to use in primary care settings. BDRI includes a combination of age and cognitive, lifestyle and cardiovascular factors. In BDRI, cognitive items are highlighted [7]. In the study of Pekkala et al. [10], cognitive performance was the most important predictor, more predictive than age or vascular factors, for subsequent dementia according to supervised machine learning method using a large number of heterogeneous factors. DSI, instead, is a combination of demographic, vascular and lifestyle factors, difficulties in instrumental activities of daily living and depressive symptoms [8]. In our study, proportion of subjects with an increased risk for dementia according to DSI was low in both cohorts. This is somehow consistent with relative low percentages of subjects with an increased risk for dementia based on DSI in earlier studies, ranging from 6 to 27% [8]. It is possible that DSI underestimates dementia risk and thereby misses asymptomatic older adults who should be targeted for cognitive screening.

In our study, slightly modified version of CAIDE and BDRI were used in our study. This may have had an impact on risk classification and total scores of indices as well as on comparison of dementia risk between the cohorts. It is also notable, that cohort comparisons cannot confirm changes in risk factors, but only differences between the cohorts. Longitudinal studies can provide more insight regarding changes in dementia risk and cognitive functioning over time.

Our next step is to compare dementia incidence between these two Finnish cohorts as well as to evaluate the prognostic value of BDRI among the cohorts.
